# First Documentation of *Exophiala* spp. Isolation in Psittaciformes

**DOI:** 10.3390/ani12131699

**Published:** 2022-06-30

**Authors:** Gonçalo N. Marques, João B. Cota, Miriam O. Leal, Nuno U. Silva, Carla A. Flanagan, Lorenzo Crosta, Luís Tavares, Manuela Oliveira

**Affiliations:** 1 Zoomarine Portugal, E.N. 125, Km 65, 8201-864 Guia, Portugal; gnmarques1205@hotmail.com (G.N.M.); miriamleal.mv@gmail.com (M.O.L.); nuno.urbani@zoomarine.pt (N.U.S.); carla.flanagan@zoomarine.pt (C.A.F.); 2 CIISA—Centro de Investigação Interdisciplinar em Sanidade Animal, Faculdade de Medicina Veterinária, Universidade de Lisboa, Av. Da Universidade Técnica, 1300-477 Lisbon, Portugal; joaobcota@fmv.ulisboa.pt (J.B.C.); ltavares@fmv.ulisboa.pt (L.T.); 3 Laboratório Associado Para Ciência Animal e Veterinária (AL4AnimalS), 1300-477 Lisbon, Portugal; 4 AEZAVEC (Avian, Exotic and Zoo Animal Veterinary Consultants), 22040 Tirol, Italy; lorenzo_birdvet@yahoo.com

**Keywords:** avian, *Exophiala* spp., fungi, Psittaciformes

## Abstract

**Simple Summary:**

The microbiomes of birds are not yet widely understood, although they are increasingly being studied. After the successful medical management of a suspected traumatic lesion in the left choana of a military macaw (*Ara militar*), in which *Exophiala* spp. was consistently reported, another 24 psittaciform birds from a Portuguese zoological collection were sampled to study the role of *Exophiala* spp. as part of the avian microbioma. Swab samples were collected from the trachea and/or choanae and proceeded for fungal isolation, in which fungal species were identified through their macroscopic and microscopic morphology. *Exophiala* spp. was identified in 60% of the birds sampled and no statistical association was found between the clinical record of the birds and the fungal isolation. This is the first report of isolation of *Exophiala* spp. in the upper airways of avian species.

**Abstract:**

Several fungi species are reported to act as opportunistic agents of infection in avian species. After the isolation of *Exophiala* spp., a dematiaceous fungal pathogen associated with a mucosal lesion in a military macaw (*Ara militar*), samples were collected from another 24 birds of the order Psittaciformes to study the possibility of *Exophiala* spp. being part of the commensal microbiota of these animals or its possible association with other clinical conditions. Swab samples were collected from the trachea and/or choanae of the birds and inoculated in Sabouraud chloramphenicol agar for fungal isolation. After incubation, fungal species were identified through their macroscopic and microscopic morphology. The presence of *Exophiala* spp. was identified in 15 of the 25 birds sampled and no statistical association was found between the clinical record of the birds and the fungal isolation. Our results suggest that *Exophiala* spp. can colonize the upper respiratory airways of psittaciform birds and has a low pathogenic potential in these animals. To the authors’ knowledge, this is the first report of *Exophiala* spp. isolation from samples of the upper respiratory tract of Psittaciformes.

## 1. Introduction

Fungal infections have a high prevalence in birds and are among the most serious systemic diseases in avian species [1]. Many factors may predispose birds to mycoses. Their highly effective respiratory system and the anatomic and physiological characteristics of their respiratory tract are some of the features that undoubtedly enable deep lung and air sac exposure to airborne threats, such as fungal spores [2,3]. The most common contamination route for spores is through inhalation, but they may also enter the organism through the digestive tract, skin, and mucosae [1].

*Exophiala* spp. are characterized by their dimorphism as well as by the formation of dark colonies, associated with the melanization of their cell wall. They are also able to form biofilms, which is considered to be an important virulence attribute [4,5]. In humans, fungal isolates of the genus *Exophiala* are considered to be opportunistic pathogens, causing eumycetoma, phaeohyphomycosis, or chromoblastomycosis [4]. The most prominent representative of the melanized fungi and the most isolated species from human infections is *Exophiala dermatitidis* (*E. dermatitidis*) [4]. Infection by *Exophiala* spp. may have a large variety of clinical manifestations, including skin, pulmonary, cerebral, and disseminated forms of infection, the latter associated with poor prognosis and high mortality rate [6,7].

The need for the present investigation emerged after the isolation of *Exophiala* spp. from a military macaw (*Ara militaris*) specimen under professional care, housed in an outdoor aviary at Zoomarine Portugal. As part of the zoological collection, this individual was included in a routine preventive medical program and had an unremarkable medical history. The bird presented signs of oral pruritus. Physical examination was clinically irrelevant except for an ulcerative linear whitish lesion, 7 mm long, in the left choana. A swab sample from the lesion was collected for mycological analysis, which was positive for *Exophiala* spp. Both topical (nystatin 300,000 U/kg, PO BID and miconazole oral gel 1 mL, PO BID) and systemic (itraconazole 10 mg/kg, PO SID) antifungal agents were used throughout the treatment of this case, but the choana lesion persisted and the oral pruritus continued, with follow-up fungal cultures remaining positive for this fungal genus. Clinical resolution was achieved after the administration of a combination of itraconazole and terbinafine (15 mg/kg, PO BID)—106 and 19 days of therapy, respectively, with no signs of an infection relapse for more than one year after the first negative result on fungal culture.

In this case report, *Exophiala* spp. may have played a pathogenic role, entering through a site of mucosa breakdown and acting as an agent of secondary local infection, thus preventing and delaying lesion healing. Given the absence of support in the clinical literature regarding *Exophiala* spp. isolation from avian species, the present study was undertaken in order to collect baseline data on several individuals from the order Psittaciformes and develop a preliminary investigation on the potential pathogenic role of *Exophiala* spp. in birds.

Microbiomes can be defined as the collective community of microorganisms which are associated with a particular environment [8]. *E. dermatitidis* has already been identified in frugivorous birds’ feces [9], but it is unclear if this yeast can be part of the avian respiratory microbiome. Reports on the composition of avian respiratory microbiome, and in particular regarding fungi, are mainly restricted to poultry and information concerning wild birds is still scarce.

## 2. Materials and Methods

### 2.1. Animals

A total of 25 birds from 12 species of the order Psittaciformes were sampled (Table 1). These birds were housed at Zoomarine in two separate zoological areas, in common or contiguous outdoor aviaries.

### 2.2. Sample Collection and Analysis

Biological samples were collected during clinical check-ups under general anesthesia with isoflurane, between October 2020 and March 2022. Check-ups were performed either on birds with an unremarkable medical history, as part of the medical preventive program of Zoomarine, or on birds with a past medical diagnosis, as part of a follow-up clinical program.

None of the birds were purposefully anesthetized for this study. Samples were collected using AMIES swab samples (VWR, Portugal) either from the trachea or the choanae, by gently introducing a sterile swab, which was twirled around and withdrawn, avoiding contact with any other surfaces. After collection, swab samples were immediately sent to the Laboratory of Mycology of the Faculty of Veterinary Medicine, University of Lisbon, for fungal isolation and identification.

All samples were inoculated in Sabouraud chloramphenicol agar (VWR, Portugal), incubated for 7 days at 28 °C and observed daily for the development of fungal colonies. The phenotypic identification of fungi was based on colony macroscopic traits and on the microscopic aspects [10]. Macroscopic characteristics, namely size, morphology, and pigmentation on both sides of the colonies were recorded. Microscopic observation was performed by lactophenol cotton blue staining wet mounts in order to examine the characteristics of hyphae and spores, and also the type of budding.

### 2.3. Statistical Analysis

Associations between *Exophiala* spp. isolation and sample site or clinical situation of the birds, and associations between *Exophiala* spp. isolation and the isolation of other fungi were assessed using Fischer exact test. Associations were considered to be significant when *p* values were less than 0.05. Statistical analysis was performed using SPSS for Windows version 28.0 (SPSS Inc., Chicago, IL, USA).

## 3. Results

**Figure 1:**
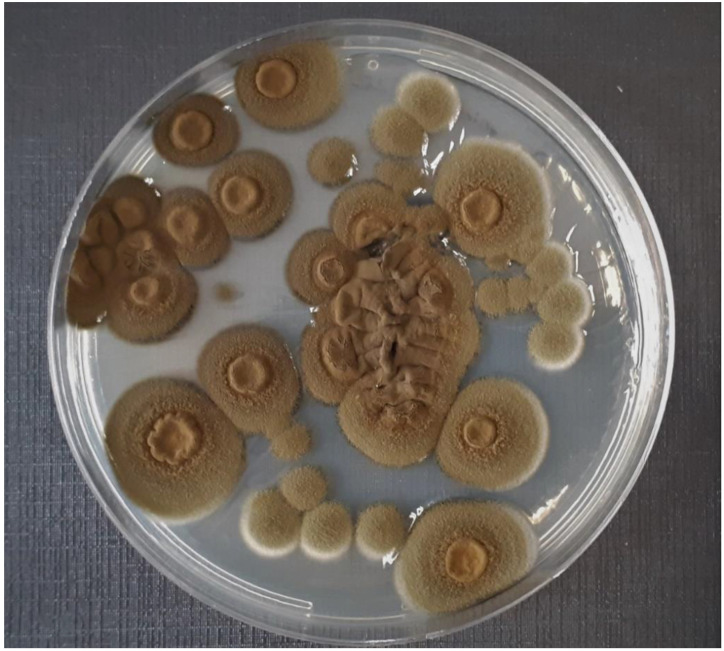
Macroscopical appearance of *Exophiala* spp. colonies, isolated from psittaciform birds, in Sabouraud chloramphenicol agar after 7 days of incubation.

**Figure 2:**
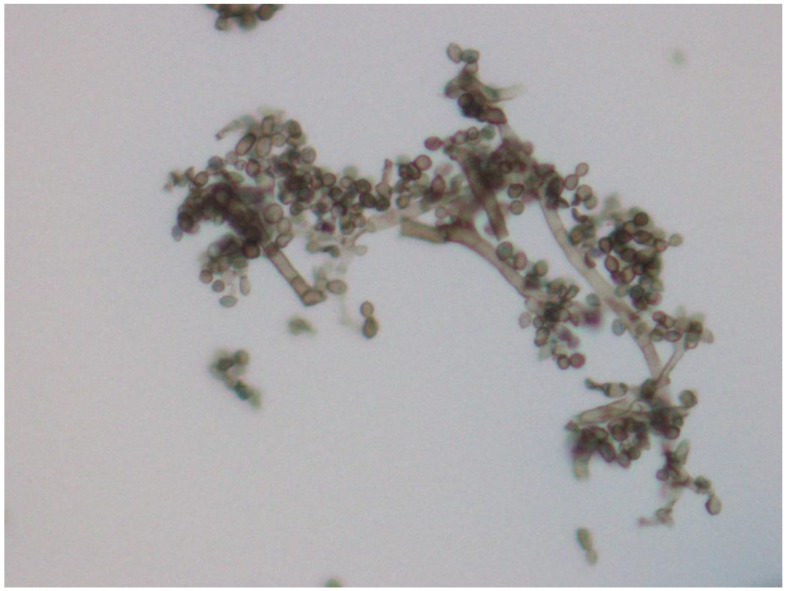
Microscopical features of *Exophiala* spp. isolated from psittaciform birds. Dark pigmented septate hyphae, together with numerous elliptical conidia. Amplification 400×.

*Exophiala* spp. was isolated from 15 (60%) of the 25 birds included in this study (Table 2). Macroscopically, the colonies ranged from an olive to a dark green color and a velvet-like appearance (Figure 1). Microscopically, septate pigmented hyphae were observed, along with budding annelids and multiple elliptical-shaped conidia (Figure 2).

Regarding the clinical situation of the psittaciform birds tested, *Exophiala* spp. was isolated from birds both with and without clinical findings (66.7% and 50%, respectively, *p* > 0.05) (Table 2). Moreover, *Exophiala* spp. infection was medically associated with clinical findings in only one of those cases (bird #21, Table 3).

Regarding the site of sample collection, despite the frequency of *Exophiala* spp. isolation from choanae being higher when compared with the tracheal samples (69.2% and 57.1%, respectively) no statistically significant difference was found (*p* > 0.05) (Table 2). Additionally, in the cases in which two samples were taken from different sites of the same bird, the results for fungal isolation and identification were identical.

The isolation of other fungi besides *Exophiala* spp. was possible in 16 (64%) of the 25 sampled animals (Table 3), including yeast species (10/16 birds), *Alternaria* spp. (6/16 birds), *Penicillium* spp. (2/16 birds), *Aspergillus* spp. (1/16 birds), and *Chrysosporium* spp. (1/16 birds). Contrarily, swabs performed on three animals resulted in no fungal isolation. No statistical associations between the isolation of *Exophiala* spp. and the concurrent isolation of other identified fungi were found (*p* > 0.05).

## 4. Discussion

To the authors’ best knowledge, this is the first report of the isolation of *Exophiala* spp. from upper airway samples of Psittaciformes. Fungi are commonly opportunistic microorganisms, which can be part of the physiological microbiota of humans and animals, including birds [11,12,13]. However, fungi may act as pathogens and there are multiple factors that may predispose an individual to fungal infections, such as immunosuppression, underlying diseases, prolonged antibiotic therapy, genetic factors, and major dirt upheavals [1,2,11,14].

Different *Exophiala* species have increasingly been associated with clinical disease in several animals, namely in invertebrates, cold-blooded animals and, to a lesser extent, in mammals [15,16,17,18,19]. The pathogenic potential of *Exophiala* spp. in avian species seems to be low when compared with other animal species, with the only available report being limited to its association with feather abnormalities in wild turkeys due to multiple aetiology mycosis [20,21].

In the present study, the prevalence of *Exophiala* spp. among the psittaciform birds tested was 60%, and no statistically significant differences were found according to the birds’ health status. In fact, only one of the birds had a clinical situation in which *Exophiala* spp. had a relevant influence on the outcome. The results obtained suggest that these birds might be colonized by these fungi. It was already hypothesized that *E. dermatitidis* could have a life cycle associated with frugivorous animals, including birds [9]. Thus, it seems that these animals can have an important role in the dissemination of *Exophiala* spp. rather than developing clinical infections. Nevertheless, additional studies are needed, namely histopathological examination of diseased animals with *Exophiala* spp. positive cultures, to better understand the possible impact of this agent.

The sources of the isolated *Exophiala* spp. and its dissemination routes among the birds studied in the present report are not known. It is important to emphasize that these dematiaceous fungi are considered to be ubiquitous in nature and can be found in warm and humid oligotrophic natural environments, being recovered, for example, from air and soil samples as showed in a recent study in a Malaysian wildlife rescue center [22]. On the other hand, *Exophiala* species have been isolated in man-made structures such as saunas [23], bathrooms [24,25,26], or even dishwashers [27,28], which replicate the natural conditions where *Exophiala* spp. is more commonly found. In this case, continuous shedding of *Exophiala* spp. in the feces of colonized birds may contribute to the fungal dispersion in the environment and the colonization of other birds living in the same aviary. Small-sized wild birds that occasionally enter the aviaries can also play a part in the dissemination of fungal species. Moreover, tropical fruits have also been proposed to be a source of *Exophiala* spp. for frugivorous animals [9], although the isolation of these fungi in other fruits has not been reported, namely from temperate climates. Finally, there are multiple reports on the association of *Exophiala* spp. and environments polluted with aromatic hydrocarbons [29,30,31,32], which seems unlikely in the presented study. Unfortunately, it was not possible to perform environmental analysis to confirm the presence of *Exophiala* spp. in the quarters where the animals were kept, which would bring more information regarding the dissemination of this agent on the premises of this zoological collection. Further research is needed to better understand these hypotheses.

Our results indicate that the upper airways of positive birds were colonized by *Exophiala* spp., thus airborne dissemination should not be excluded. Dust particles carrying *Exophiala* spp. could be inhaled by the birds leading to the colonization of their respiratory tract. The colonization of the airways by *E. dermatitidis* in humans is associated with cystic fibrosis patients, though its involvement in lung disease progression it is not clear, nor whether this colonization is the result of the impairment of normal respiratory tract function [4]. Still, considering the results from our study, *Exophiala* spp. do not seem to be an important respiratory pathogen of avian species from the order Psittaciformes.

In birds, the most frequently isolated fungi are part of the genera *Aspergillus*, which is mostly associated with infections of the respiratory system, and *Candida*, associated with gastrointestinal infections [1,2]. In the present study, fungi other than *Exophiala* spp. were isolated, most frequently yeast species with a morphology compatible with *Candida* spp. These results are similar to those reported previously in a study focusing on the fungal microbiota of the trachea of birds in a wildlife rehabilitation center in Spain, in which more than 65% of all yeasts isolated were identified as *Candida* spp. [12].

In this report, we present the results of the isolation of fungi from the upper airways of psittaciform birds of a Portuguese zoological collection after the successful clinical management of a military macaw with a mucosal lesion, in which *Exophiala* spp. was consistently isolated. The clinical management of this uncommon fungal infection was challenging and a long-term course of antifungal agents—more than three months of itraconazole therapy, adding terbinafine to the multimodal treatment for 19 days—was needed. Accordingly, the literature supports the eventual need for long-term periods of treatment in various fungal infections, along with the importance of a continuous follow-up given the possibility of disease reactivation [33,34]. This clinical case was the trigger to assess the prevalence of *Exophiala* spp. in these birds, also providing new information regarding the ecology of dematiaceous fungi.

Timely diagnosis of fungal infections is crucial to promptly initiate treatment and improve the overall prognosis [33]. Moreover, clinicians are advised to entail a critical thinking posture when analyzing mycological results. It is important to bear in mind that many fungi species are ubiquitous, thus a positive culture result does not imperatively mean there is an infectious process in development. The possibility of environmental contamination should be considered, and cytological examinations should be conducted. Moreover, the microbiomes of birds are not yet widely understood, although they are increasingly considered a topic of study. Further species-specific investigations on this subject are still necessary, especially concerning the role of fungi as part of the overall avian microbiome.

Studying tropical bird communities is of utmost importance, in terms of ecology and conservation, yet difficulties are imposed in the design and development of such research studies. In the present report, choanae and tracheal samples were collected during routine or follow-up check-ups under general anaesthesia. In a wildlife context, such sampling involves avian capturing in the first place, which can lead to animal distress, pain and cause injuries to the birds which can ultimately result in death. Furthermore, the use of general anaesthesia can be impractical on those circumstances, impairing the possibility of collecting samples without additional risks during the procedure. The development of alternative respiratory tract sample collection methods in wildlife populations are needed in order to avoid such setbacks. The literature suggests the use of non-invasive and practical methods of fecal sample collection as substitute procedures when considering microbiome research, whereas oral and respiratory sampling methods are mainly performed in animals submitted to wildlife rehabilitation centres [12,35,36,37].

Despite the impediments in the development of studies on wild birds’ microbiomes, these are considered to be essential since captivity is known to alter their composition [8]. Nevertheless, though captive (ex situ) populations of wild birds are not exposed to the same conditions as in situ populations, they can be used as an alternative to better understand the microbiomes of these animals. Additionally, these ex situ populations can also highlight the potential impacts of the human activity on in situ populations regarding conservation objectives, since the biodiversity of those microbiomes is considered to be a crucial component in wildlife management practices [38].

## 5. Conclusions

Birds can be naturally exposed to fungi without developing mycoses. However, it is important to note that early diagnosis and management of systemic fungal infections can be challenging, hence the importance of maintaining a thorough medical preventive program in birds under human care. This study was undertaken after a clinical case in which *Exophiala* spp. was isolated, re-examining the fact that although fungi are seldom considered infectious agents in immunocompromised birds, they can also affect immunocompetent individuals. This is the first report of isolation of *Exophiala* spp. in the upper airways of avian species, suggesting the possibility of colonization of their respiratory tract.

## Figures and Tables

**Table 1:** Identification of the psittaciform birds sampled.

Bird	Species	Year of Birth	Gender
1	*Agapornis fischeri*	Unknown	Male
2	*Agapornis nigrigenis*	2019	Unknown
3	*Amazona aestiva xanthopteryx*	2005	Female
4	*Amazona autumnalis*	Unknown	Male
5	*Amazona autumnalis*	2000	Female
6	*Anodorhynchus hyacinthinus*	2018	Female
7	*Anodorhynchus hyacinthinus*	2019	Male
8	*Ara ararauna*	1997	Female
9	*Ara ararauna*	1998	Female
10	*Ara ararauna*	1999	Female
11	*Ara ararauna*	2004	Male
12	*Ara ararauna*	2005	Female
13	*Ara ararauna*	2008	Female
14	*Ara ararauna*	2016	Male
15	*Ara chloropterus*	1991	Female
16	*Ara chloropterus*	2016	Female
17	*Ara chloropterus*	2016	Male
18	*Ara macao*	1991	Female
19	*Ara macao*	2015	Female
20	*Ara militaris*	2012	Female
21	*Ara militaris*	2016	Female
22	*Cacatua alba*	1997	Male
23	*Eclectus roratus*	Unknown	Female
24	*Eclectus roratus polychloros*	2016	Female
25	*Psittacus erithacus*	Unknown	Female

**Table 2:** Distribution of *Exophiala* spp. isolation frequencies regarding clinical situation of the birds and sampling site.

Clinical Situation	Positive (%)	Negative (%)	Total	*p* Value
Unremarkable	5 (50%)	5 (50%)	10	>0.05
With clinical findings	10 (66.7%)	5 (33.3%)	15	
Total	15	10	25	
Sampling site				
Choanae	9 (69.2%)	4 (30.8%)	13	>0.05
Trachea	8 (57.1%)	6 (42.9%)	14	
Total	17	10	27	

**Table 3:** Isolation of *Exophiala* spp. and other fungi in samples of psittaciform birds, with respective clinical situation.

Bird	Sampling Site	Isolation of *Exophiala* spp.	Isolation of Other Fungi	Clinical Situation
1	Choanae	Negative	-	Sudden death associated with intestinal disease
2	Choanae	Negative	Yeast species	Unremarkable
3	Trachea	Positive	Yeast species	Unremarkable
4	Choanae and trachea	Positive	*Alternaria* spp. *Aspergillus* spp.	Mild tracheal stenosis
5	Choanae	Positive	Yeast species *Chrysosporium* spp.	Occasional respiratory signs
6	Trachea	Negative	Yeast species	Unremarkable
7	Choanae	Negative	-	Unremarkable
8	Choanae	Positive	-	Arthrosis
9	Trachea	Negative	*Penicillium* spp.	Occasional respiratory signs
10	Choanae	Positive	Yeast species	Occasional respiratory signs. Mild hydropericardium and dilated right ventricle
11	Trachea	Positive	*Alternaria* spp.	Poor feathering
12	Choanae	Negative	*Alternaria* spp.	Suspected mild cardiomegaly
13	Trachea	Negative	*Alternaria* spp. *Penicillium* spp.	Unremarkable
14	Trachea	Negative	-	Unremarkable
15	Choanae	Positive	*Alternaria* spp. Yeast species	Third-degree atrioventricular block
16	Trachea	Positive	-	Poor feathering
17	Choanae	Positive	-	Unremarkable
18	Choanae	Positive	-	Arthrosis and psittacid herpesvirus positive
19	Trachea	Positive	Filamentous fungi	Unremarkable
20	Trachea	Negative	*Alternaria* spp. Yeast species	Miocarditis
21	Choanae and trachea	Positive	Yeast species	Delayed healing of traumatic lesion. Improvement after antifungal therapy.
22	Trachea	Positive	-	Unremarkable
23	Choanae	Positive	Yeast species	Sudden prostration and leukocytosis—improved quickly after antibiotic therapy. Poor feathering
24	Trachea	Negative	Yeast species	Poor feathering
25	Trachea	Positive	-	Unremarkable

## Data Availability

Not applicable.
